# Characteristic Volatiles and Cultivar Classification in 35 Apple Varieties: A Case Study of Two Harvest Years

**DOI:** 10.3390/foods11050690

**Published:** 2022-02-25

**Authors:** Xinye Wu, Jinfeng Bi, Marie-Laure Fauconnier

**Affiliations:** 1 Institute of Food Science and Technology, Chinese Academy of Agricultural Sciences, Key Laboratory of Agro-Products Quality and Safety Control in Storage and Transport Process, Ministry of Agriculture and Rural Affairs, P.O. Box 5109, Beijing 100193, China; wuxinye@caas.cn; 2 Laboratory of Chemistry of Natural Molecules Gembloux Agro-Bio Tech, University of Liege, Passage des Déportés 2, 5030 Gembloux, Belgium; marie-laure.fauconnier@uliege.be

**Keywords:** apple, HS-SPME-GC-MS, volatile, multivariate analysis, odor-active compounds

## Abstract

A large number of apple varieties (35) from the same germplasm were investigated over two consecutive harvest years. A total of 39 volatile compounds were identified by HS-SPME-GC-MS, and quantified by external standards. Principal component analysis was applied to study the relationship between varieties and volatiles. To obtain better discrimination, 23 of 35 apple varieties were classified into four cultivars and good classification could be observed by partial least squares discriminant analysis. Ethyl 2-methylbutyrate, 2-methyl-1-butanol, *Z*-3-hexenyl acetate, *E*-2-hexen-1-ol, linalool and dodecanol were the most important variables to discriminate apple cultivars. Based on the volatile concentration and thresholds, ethyl 2-methylbutyrate, hexanal, 1-hexanol, *E*-2-nonenal and linalool were the critical characterized odor-active compounds among 35 apple varieties over two harvest years. From the present work, seasonal effects greatly influenced the formation of volatiles.

## 1. Introduction

Aroma is an apple quality attribute consisting of not only one critical component but a complex mixture of volatiles which relies on volatile compounds, their concentrations and odor thresholds. Currently, more than 300 volatiles have been identified in apples [1]. Most of the volatiles are classified into esters (78–92% of total volatiles), alcohols (6–16% of total volatiles), aldehydes and ketones [2]. However, the compositions and concentrations of volatiles are affected by many factors, including stage of maturity [3,4,5], storage conditions [3], climates [6] and terroir effect [7]. For instance, aldehydes are dominant in immature apples. However, the concentrations of aldehydes decrease during the apple ripening period, while esters and alcohols become the main volatiles with their concentrations increasing [8]. Additionally, apple variety is another key factor that has a great influence on volatiles. Studies on various varieties reveal many differences both in volatiles’ composition and concentration [4,9,10,11].

There are many typical methods of volatile extraction, including purge and trap, dynamic headspace, liquid–liquid extraction and solvent assisted flavor evaporation. The headspace solid phase micro-extraction (HS-SPME) coupled with gas chromatography–mass spectrometry (GC-MS) technique is frequently applied to fruit [7,9,10,12,13,14] volatile detection, due to its advantages of being easy, convenient and environmentally friendly.

Multivariate analysis has been employed to discriminate and classify fruit based on volatile profiles in many studies. Principal component analysis (PCA) is a useful tool to distinguish the differences among samples and study the relationship between samples and volatiles. It is frequently applied to fruit, including apple [9,10,12], peach [13] and kiwifruit [15]. However, PCA cannot be applied to sample classification. Although PCA may indicate clear separation by a bi-dimensional plot, a complementary technique is also demanded in parallel [16]. Partial least squares discriminant analysis (PLS-DA) is one of the widely used methods to classify different groups of samples. This pattern recognition method has been previously studied in apple [12,17] and other fruits [15,18].

Although volatile concentrations can reflect the contribution to volatile compounds, not all volatiles contribute to the aroma. Odor threshold values should also be taken into account. For example, although the amounts of some specific volatile compounds were low, they may have a greater contribution to the aroma due to their low odor threshold. Komthong et al. [19] reported methyl 2-methylbutyrate, ethyl 2-methylbutyrate, isobutyl acetate, ethyl butanoate, isopentyl formate, butyl acetate, hexyl acetate, hexanal and hexanol were the odor-active compounds in Fuji apple. Similar results were found by Ortiz et al. [5]; hexyl acetate, hexyl propanoate, hexyl 2-methylbutyrate, ethyl 2-methylbutyrate, 2-methylbutyl acetate and butyl 2-methylbutanoate were considered the odor-active compounds in Golden Reinders apples.

There were several seasonal factors that affect volatile emissions such as temperature and precipitation. High temperature may inhibit enzyme activity, and water deficit limits the volatile potential [20]. However, excessive rain may also reduce the content of volatiles [21]. There was no consistent conclusion on whether the season has an influence on volatiles that is stronger than cultivar differences. Zarid et al. [22], Schwieterman et al. [23], and Noriega et al. [21], demonstrated that seasonal effects were stronger than cultivar effects on melon and strawberry aromas. However, Giannetti et al. [17], demonstrated there was no seasonal effect on ancient apple cultivars, which could be explained by ancient apple cultivars being more resistant to adverse climatic changes.

In the present study, 35 apple varieties have been investigated by HS-SPME-GC-MS from two consecutive harvest years with the following aims: The first aim was to identify volatile compounds among 35 apple varieties, which were important for obtaining the volatile profiles. The second aim was to compare the similarities and differences between varieties and years, using multivariate analyses. The third aim was to identify the critical odor-active compounds based on the OAV results over two harvest years, among all the varieties. It was also worthy to investigate whether the season greatly affected apple volatiles. Through the present study, we hope to provide new insights into identifying odor-active compounds and cultivating varieties with strong apple aromas.

## 2. Materials and Methods

### 2.1. Materials

A total of 35 varieties of apple samples were collected from the National Apple Germplasm Resource, Institute of Pomology (Liaoning Province, China), Chinese Academy of Agricultural Sciences (CAAS) over two consecutive harvest years. To ensure the consistency of each variety, the apples had been randomly picked from the same trees for two years. The harvest dates were the same for two years, which are listed in Table 1. A total of 5 kg of each variety were randomly picked from three apple trees with similar fruit weights, tree shape and growth conditions. At the same time, all the apples were free from visible external damage, including decay, rot disease and worm holes. The detailed information of 35 apple varieties are shown in Table 1. After harvest, the apples were immediately transported to the Institute of Food Science and Technology (Beijing, China), CAAS. In order to keep the same stage of maturity, the starch–iodine index was examined by the method of Blanpied & Silsby [24]. When the apples reached the same stage of maturity, they were washed, cored and sliced along the largest transverse diameter. In order to lower the loss of volatiles, all the apples were treated with liquid nitrogen after pre-treatment and stored in a −80 °C refrigerator, and all tests were completed within one month.

### 2.2. Chemicals

The 39 external standards and 1 internal standard were purchased from 4 different companies. For example, 2-methyl-1-butanol (≥98.0%), 1-hexanol (≥98.0%), butyl propionate (≥98.5%), pentyl acetate (≥98.5%) and naphthalene (≥99.0%) were provided by J&K Chemical Ltd. (Beijing, China). Butyl octanoate (>99.0%), hexanal (>98.0%), ethyl butyrate (>98.0%), butyl acetate (>99.0%), *E*-2-hexenal (>97.0%), ethyl 2-methylbutyrate (>98.0%), D-limonene (>99.0%), 1-heptanol (>98.0%), butyl butyrate (>99.0%), ethyl hexanoate (>99.0%), hexyl acetate (>99.0%), 2-ethylhexanol (≥99.0%), 1-octanol (>99.5%), propyl hexanoate (>98.0%), linalool (>96.0%), nonanal (>95.0%), hexyl propionate (>98.0%), hexyl hexanoate (>98.0%), hexyl butyrate (>98.0%) and 2-octanol (>98.0%) were obtained from TCI Development Co., Ltd. (Shanghai, China). *Z*-3-hexenyl acetate (≥97.0%), pentyl butyrate (≥98.0%), hexyl 2-butenoate (≥95.0%), hexyl octanoate (≥97.0%), *E*-2-hexen-1-ol (≥95.5%), butyl hexanoate(≥98.0%), ethyl octanoate (>99.0%), anethole (>98.0%), dodecanol (>99.0%), pentyl acetate (>98.0%) and *E*-2-nonenal (≥95.0%) were gained from H&K Flavor (Shanghai, China). 6-methyl-5-hepten-2-one (>98.5%), butyl 3-methylbutanoate (≥98.0%), hexyl 2-methylbutanoate (≥98.5%) and estragole (≥98.0%) were obtained from Yuanpeng Flavor & Fragrance Group Co., Ltd. (Tianjin, China). *n*-alkane series (C_7_–C_30_) were purchased from Sigma-Aldrich, St. Louis, MO, USA. All the chemicals mentioned above were GC grade. In addition, naphthalene was in crystal flake form, and was dissolved in methanol before use, others were in solution form. Other chemicals, such as sodium chloride and methanol were purchased from Sinopharm Group Chemical Reagent Co., Ltd. (Shanghai, China).

### 2.3. HS-SPME

The fiber materials, water content added into vial, sample amount, the concentration of sodium chloride, extraction time and extraction temperature had been already optimized in preliminary experiments. Apple samples were taken out from the freezer and one piece randomly selected from 10 apples in each variety. The apples were cut into small pieces without being defrosted and mixed well. Four grams of apple pieces were placed into a 20 mL vial with 2 mL sodium chloride solution (0.3 g/mL, in deionized water). At the same time, 100 μL 2-octanol (0.822 mg/L, in methanol) was placed into the vial as an internal standard. The aluminum cap was closed immediately with a polytetrafluoroethylene/silicone septum to avoid apple oxidation. After being thawed at room temperature, the samples still remained the original apple color.

A 65 μm polydimethylsiloxane/divinylbenzene (PDMS/DVB) fiber was selected to perform the SPME based on preliminary experiments. The fiber was conditioned prior to analysis based on the recommendations from the manufacturer (Supelco, Bellefonte, PA, USA), at 250 °C for 30 min. The extraction was applied by an AOC-5000 auto sampler (Shimadzu, Japan) at a stirring speed of 250 rpm. The incubation and extraction conditions were set at 35 °C for 20 min and 40 min, respectively. The fiber was thermally desorbed at 200 °C for 3 min, and was fully desorbed with no carry-over of the analyte.

### 2.4. GC-MS Analysis

A QP2010Plus (Shimadzu, Japan) was used for GC-MS analysis. A DB-WAX column (30 m × 0.25 mm × 0.25 μm, Agilent J&W GC Column, USA) with a constant helium flow of 1 mL/min was used. Injection of blanks (without apple sample) was performed at the beginning and between each apple variety to eliminate noise. The temperature of the injector and transfer line was kept at 250 °C. The oven temperature program began at 40 °C and was kept constant for 3 min. It was then increased to 120 °C at the rate of 5 °C/min, and from 120 °C to 230 °C at the rate of 10 °C/min and kept at 230 °C for 5 min. Mass spectra source temperature was set as 200 °C, with 70 eV. The scan range was between 35–500 amu with a 3 min solvent delay.

### 2.5. Compound Qualification and Quantification

The volatiles were identified by comparing retention time and mass spectra with the NIST11 library initially. A series of *n*-alkanes (C_7_–C_30_) were analyzed by GC-MS under the same conditions as mentioned in Section 2.4. The identified compounds were then reconfirmed by calculating the linear retention index with standards [25].

A total of 39 compounds were used as external standards to quantify apple volatiles. Selected ion monitoring (SIM) data acquisition mode was applied due to its high sensitivity, accuracy and detection limits. Three specific ions were selected for each compound the most abundant one for a quantification ion, and the other two for identification. All standards were dissolved in methanol. According to preliminary experiments, the solutions were diluted by 1, 100, 200, 400, 800, 1600 and 3000 times based on their responses in GC-MS. Both 100 μL internal and mixed external standards were added into the vial with 2 mL water and run in the test. The concentrations of each identified volatile compound were calculated by standard curves Y = aX + b. Y represented the ratio of the peak area of the standard to that of the internal standard. And X stood for the weight of the standard in grams. All the experiments were carried out in triplicate.

### 2.6. Calculation of OAV

Odor active value (OAV) is the ratio of the concentration of each compound to its detection odor threshold. If the OAV was greater than 1, it was considered to contribute to the odor and was regarded as an odor-active compound.

### 2.7. Statistical Analysis

In the case of PCA, the observations and variables were apple varieties and volatile compounds, respectively. The input values were volatile concentrations. The results were displayed by score plots and loading plots. The score plot explains the differences/similarities among different apple varieties. Loading plots investigated which volatile compounds had higher contributions to differences/similarities. PLS-DA was developed to separate the samples into classes using prior knowledge. R^2^X and R^2^Y were the percentages of variation of X and Y to explain the model. Q^2^ was the predictive ability [26]. Negative values of Q^2^ revealed a lower risk of overfitting, illustrating the robustness of the model [12]. The variable importance in the projection (VIP) value reflected the contribution used to classify the sample into groups. A higher VIP value (>1) was most relevant for explaining Y variables and contributed more to distinction among samples.

Based on the literature [13,27,28], reported parentages or origins of apples are listed in Table 1. The information for 12 apple varieties were unknown and were excluded. The remaining 23 apple varieties were classified into 4 groups (Table 1), which were *cultivar.* Golden Delicious (c. GD), *cultivar.* Jonathan(c. JT), *cultivar.* Ralls (c. RA) and *cultivar.* Fuji (c.FJ). PCA and PLS-DA were both performed by SIMCA (version 14.1, Umetrics, Sweden).

## 3. Results and Discussion

### 3.1. Volatile Compounds Analysis

**Figure 1:**
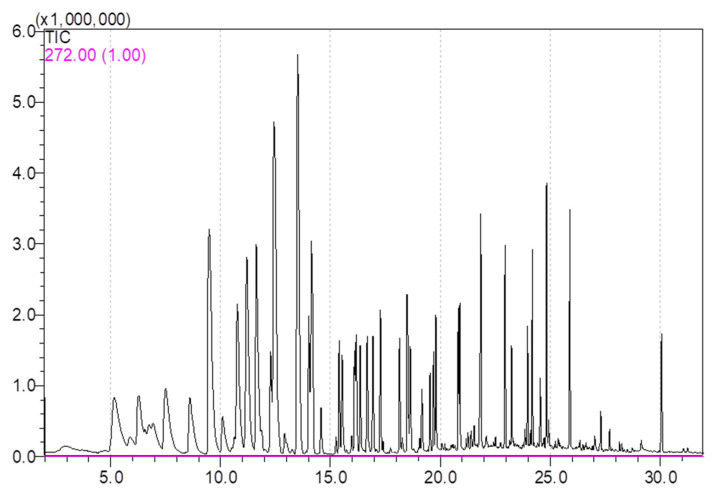
Total ion current (TIC) chromatogram graph for 1 internal standard and 39 external standards.

According to the retention time, retention index and qualitative masses (*m*/*z*), 39 volatile compounds were identified, including 22 esters, 8 alcohols, 4 aldehydes, 1 ketone, 1 terpenoid and 3 others (estragole, naphthalene and anethole) in all apple varieties over two years. All the volatiles had been reported in apple or apple juice in previous studies [1,4,7,9,26,29]. The volatiles were then quantified by external standards, calculated with standard curves. The total ion current (TIC) chromatogram graph for internal and external standards is shown in Figure 1. Detailed apple volatile concentrations for 1st year and 2nd year were in the Supplementary Materials Tables S1 and S2.

**Figure 2:**
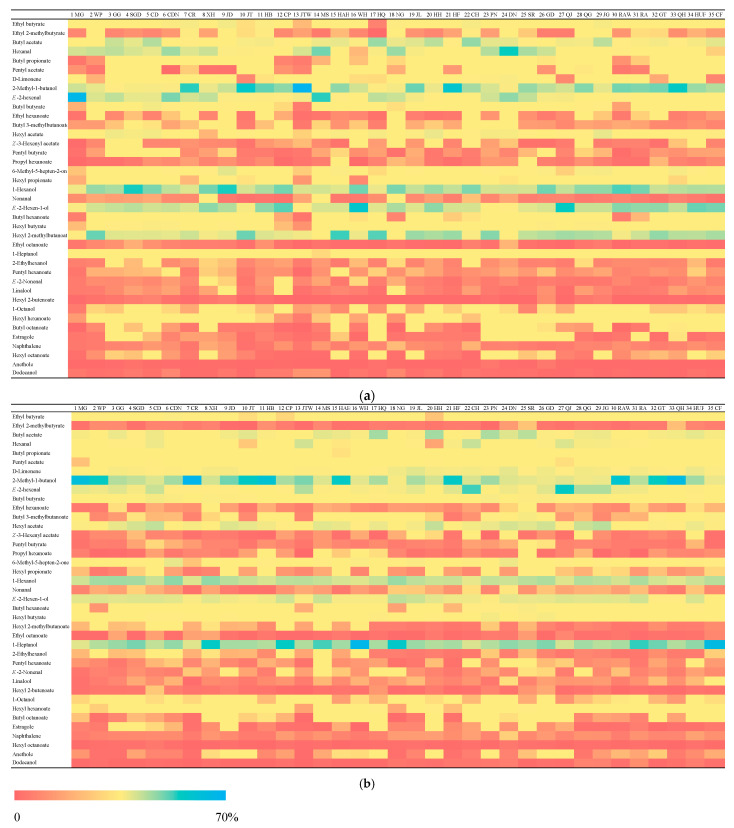
Heatmap displaying the percentages of 39 volatile compounds among 35 apple varieties in the first year (**a**) and the second year (**b**). The colors from red to blue represent the percentages from low to high for each variety. The apple cultivar abbreviation codes are MG-Miguo, WP-White Pippin, GG-Grimes Golsen, SGD-Stark Spur Golden Delicous, CD-Cloden, CDN-Cardinal, CR-Calville Rouge, XH-Xinhua, JD-Jonagold, JT-Jonathan, HB-Hadi Bolaite, CP-Clapp, JTW-Jonathan (New), MS-Mutsu Spur, HAH-Hahong, WH-Wenhong, HQ-Hongqiaowang, NG-Ningguan, JL-Judeline, HH-Huahong, HF-Hanfu, CH-Changhong, PN-Pinova, DN-Dounan, SR-Starkrimson, GD-Gloden Delicious, QJ-Qiujin, QG-Qinguan, JG-Jiguan, RAW-Ralls (weeping), RA-Ralls, GT-Gongteng, QH-Qihu 7, HUF-Huafu and CF-Changfu 2.

In order to compare and interpret the volatile compounds visually within 35 apple varieties, the quantified data were converted to percentages for both years (Figure 2). The percentages were counted by dividing the concentration of each compound by the total concentration for each variety, which are revealed by different colors. Butyl acetate, hexanal, 2-methyl-1-butanol, E-2-hexenal, 1-hexanol, E-2-hexen-1-ol, hexyl 2-methylbutanoate and 1-heptanol had relatively higher percentages in all varieties for both years, which are shown with the percentages near 70%. In other words, the contents for these compounds are dominant in apples. These compounds are all identified in the literatures [9,10] among different apple varieties.

It was noted that the concentrations of butyl acetate, 2-methyl-1-butanol and 1-hexanol were all at high levels, which is consistent with the literature. However, 2-methyl-1-butanol was not an original compound in apple, but is generated by processing, especially when mashing the apples. Although the apples were not processed, they were cut into small pieces during the extraction procedure, which may have affected formation. 2-methyl-1-butanol may form by transamination and decarboxylation of leucine and isoleucine, respectively [30].

Although hexanal, E-2-hexenal and E-2-hexen-1-ol reached higher concentrations in this study, this was not in agreement with the literature [9]. This is probably because these compounds were highly dependent on variety, as proven by Nikfardjam and Maier [31]. For instance, ascorbic acid content in apples had been reported as one of the factors that may have effects on their concentrations [32]. Moreover, all of these three compounds were generated from fatty acid metabolism. The precursor substance for hexanal and 1-hexanol is linoleic acid (C18:2). Hexanal is formed initially by the lipoxygenase (LOX) pathway, and then hexanol is produced by alcohol dehydrogenase (ADH) [8]. At the same time, hexanal could also yield E-2-hexenal from linolenic acid. Furthermore, E-2-hexen-1-ol could be produced from E-2-hexenal via ADH. Because the concentrations for 1-hexanol, hexanal, E-2-hexenal and E-2-hexen-1-ol were all at high levels in this study, it implies that the precursor contents for linoleic acid and linolenic acid may also be at high levels in these varieties.

The percentages of ethyl 2-methylbutyrate, Z-3-Hexenyl acetate, propyl hexanoate, ethyl octanoate, E-2-nonenal, linalool, hexyl 2-butenoate, naphthalene, hexyl octanoate, anethole and dodecanol were lower among all the varieties over two years, as shown by the red color.

Ethyl 2-methylbutyrate could be produced from isoleucine by isoleucine catabolism, which shares the same precursor substance as 2-methyl-1-butanol. Rowan et al. [33] treated Granny Smith apple skin with deuterium-labeled isoleucine and found ethyl 2-methylbutyrate was mostly formed. Since the 2-methyl-1-butanol was high yield, it may lack isoleucine for ethyl 2-methylbutyrate generation, thus affecting the concentration. Moreover, Yahia et al. [34] pointed out that ethyl 2-methylbutyrate was a postharvest-induced volatile, and that the concentration would be increased in apples ripened off the tree, and after a long time in cold storage. However, since all the apple samples were treated when reaching the same stage of maturity as measured by the starch-iodine index, and stored without a long time in cold storage, the postharvest-induced generation effect may be negligible.

The precursor substance for Z-3-hexenyl acetate was Z-3-hexenal, which was produced from linolenic acid (C18:3) by the LOX pathway. However, Z-3-hexenal could not only form Z-3-hexenyl acetate through ADH and alcohol acyltransferase (AAT), but could also generate E-2-hexenal via E-2-enal isomerase [8]. In other words, Z-3-hexenyl acetate and E-2-hexenal share the same precursor substance; Z-3-hexenal. Contreras et al. [35] demonstrated that the lack of linolenic acid may cause the absence of Z-3-hexenal, E-2-hexenal and E-2-hexen-1-ol. Nevertheless, the concentrations for E-2-hexenal and E-2-hexen-1-ol were relatively high for all varieties over two years, which may indicate the content of linolenic acid is high. Accordingly, Z-3-hexenyl acetate concentrations were contrasted with E-2-hexenal. It is worth noting that LOX is highly positively related to generation of these “green note” compounds (hexanal, E-2-hexenal and E-2-hexen-1-ol) [5]. The activity of LOX is increased during apple ripening in various apple varieties [8,35].

Naphthalene had been reported in apple [29], apple cider [36], mango [37], pear [38] and melon [22]. Coincidentally, Zhu et al. [29] had identified naphthalene in apples, where the apples were harvested from the same orchard as this study. There may be several reasons that cause the presence of naphthalene. Naphthalene-derived compounds, like 1-naphthaleneacetic acid, are usually used as plant growth regulators to increase yield in fruit, such as apple, pears and grapes [39]. There may be residues from the use of related regulators in field management. Some authors indicated naphthalene may be generated from the thermal degradation of β-carotene [40,41]. Moreover, some authors demonstrated that mild thermal treatment like simultaneous distillation and extraction caused the formation of naphthalene in cashew apple fruit. When the extraction temperature was below 37 °C, no naphthalene was detected [42]. In this study, although the extraction temperature was 35 °C, the extraction time was longer. Whether this affects the formation of naphthalene is unclear. The lower concentration of naphthalene may be because it was a residue from field management, β-carotene degradation products, or formed in the extraction process. Because of this uncertainty naphthalene was not excluded in the following analysis.

As for the comparison between the two years among the 39 volatiles, some of the volatiles indicated similar trends. For example, the concentration percentages for pentyl acetate, D-limonene, hexyl acetate, 1-heptanol, hexyl 2-butenoate and anethole in the first year were lower than the second year for most apple varieties. Especially for D-limonene, 1-heptanol and anethole, where the concentration percentages were increased with time for all varieties. In contrast, some of the volatiles were decreased, including ethyl 2-methylbutyrate, hexanal, hexyl propionate, E-2-hexen-1-ol, hexyl 2-methylbutanoate and hexyl octanoate. In Figure 2, hexyl 2-methylbutanoate was the most obvious volatile (blue color) in all varieties.

These compounds may be greatly affected by the environment, excluding the soil environment and the field management. Because all of the apples were from the same orchard and followed regular agronomical practice, the differences probably came from seasonal effects such as precipitation, temperature and light intensity [8] over these two years. Climate change may have effects on the formation of fatty acids and amino acids, thus impacting on precursor substances for those volatiles and their concentrations. However, this was in contrast with the work of Giannetti et al. [17] on ancient apples.

### 3.2. Multivariate Analysis

#### 3.2.1. PCA

**Figure 3:**
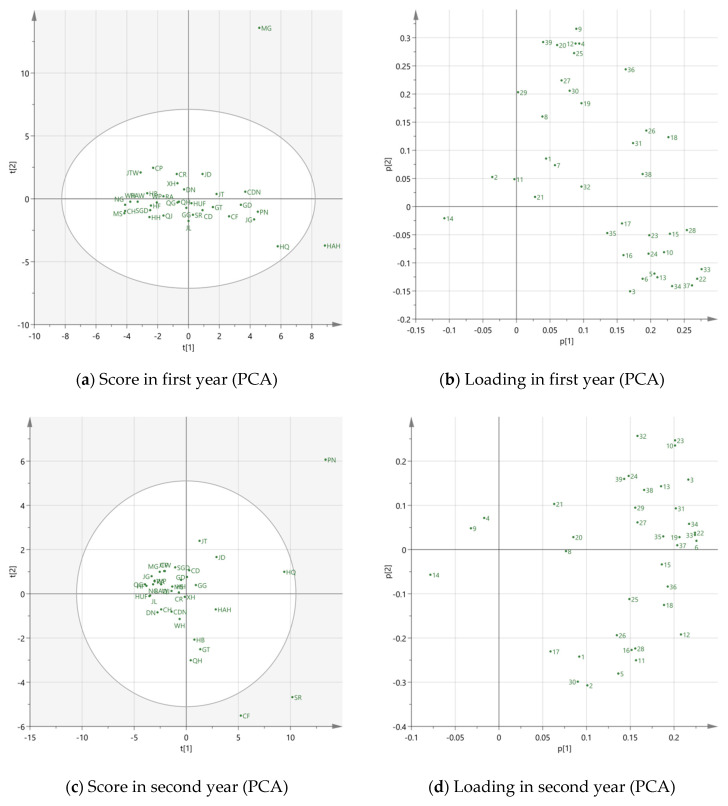

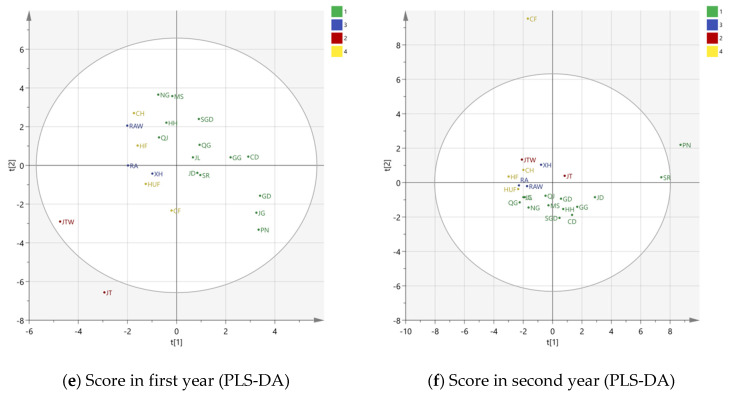
PCA and PLS-DA scores and loading plots over two years. (**a**) PCA score plot in first year; (**b**) PCA loading plot in first year; (**c**) PCA score plot in second year; (**d**) PCA loading plot in second year; (**e**) PLS-DA score in first year; (**f**) PLS-DA score in second year. In Figure 3b,d, 1 to 39 represent the volatiles detected in HS-SPME-GC-MS, detailed information is listed in Table 2. The apple cultivar abbreviation codes are described in Table 1. In Figure 3e,f, 1 = *cultivar.* Golden Delicious, 2 = *cultivar.* Jonathan, 3 = *cultivar.* Ralls and 4= *cultivar.* Fuji.

In order to reduce the number of dimensions, PCA was employed to investigate the relationship between apple varieties and volatile compounds, and which were the most important volatiles for different apple cultivars. The correlation matrixes are in the Supplementary Materials (Tables S3 and S4). The PCA results were shown in Figure 3, including the score plots and loading plots over two years.

In the first year, the eigenvalues of eight principal components (PCs) were higher than one, and the cumulative contribution was 82.18%. The first two PCs were accounted for at 25.61% and 19.17%, respectively, explaining 44.78% of the total variance in the data set. It can be seen from Figure 3a, most of the apple varieties were closely located, which indicated they were similar from a volatile point of view and may be difficult to distinguish. However, MG and HAH, located in the positive region of PC1, were distinctly separated from most apple varieties, which demonstrates that they are highly different from others. Combined with the loading plot in Figure 3b, hexanal (4), E-2-hexenal (9), butyl 3-methylbutanoate (12), hexyl propionate (18), nonanal (20), ethyl octanoate (25), 2-ethylhexanol (27), naphthalene (36) and dodecanol (39) were the main contributors for MG, leading to these differences. It was worth noting that most of these compounds were aldehydes and alcohols, which implied that MG was more related to aldehyde or alcohol like variety. As for HAH, butyl propionate (5), propyl hexanoate (16), butyl hexanoate (22), 1-heptanol (26), pentyl hexanoate (28) and hexyl octanoate (37) were the main characterized volatile compounds. Because most of these compounds are esters, HAH may be an ester like apple variety.

In the second year, there were also eight PCs. A total 53.23% of the variance could be explained by the first two PCs, which occupied 43.1% and 10.13% respectively. PN, SR and CF were positioned away from other apple varieties, which revealed that the differences in volatiles were great (Figure 3c). PN was highly influenced by butyl acetate (3), butyl butyrate (10), butyl hexanoate (22), hexyl butyrate (23), hexyl 2-methylbutanoate (24), 2-ethylhexanol (27), 1-octanol (32), butyl octanoate (34) and dodecanol (39). The main contributed volatiles of SR were ethyl 2-methylbutyrate (2), ethyl hexanoate (11), pentyl butyrate (15), propyl hexanoate (16), hexyl propionate (18), butyl hexanoate (22) and pentyl hexanoate (28), which were positively correlated to PC1. Moreover, all of them were esters, implying that SR might be an ester like variety. CF was in the negative area of PC2 and near the axis of PC1. It was highly associated with butyl propionate (5), butyl 3-methylbutanoate (12), 1-heptanol (26), linalool (30) and naphthalene (36).

It should be noticed that most of the contribution of volatile compounds mentioned above was not at a high percentage level, excluding butyl acetate (3), hexanal (4), E-2-hexenal (9), hexyl 2-methylbutanoate (24) and 1-heptanol (26). It may be concluded that the low volatile concentration percentages such as propyl hexanoate (16), ethyl octanoate (25), linalool (30), naphthalene (36), hexyl octanoate (37) and dodecanol (39), may have a greater influence on variety discrimination. To conclude, the two year results for the apple varieties MG, HAH, PN, CF and SR did not reveal similar positions in score plots and locate outside the confidence area (95%). These 5 varieties may be more sensitive and unstable with environmental changes. In general, PCA was not an effective method to distinguish apple varieties based on the volatile profiles.

#### 3.2.2. PLS-DA

Since the PCA did not show a clear separation of apple samples, PLS-DA was applied. Unlike the unsupervised method in PCA, the supervised PLS-DA method was used to sharpen the separation between groups of observations by rotation of PCA components in a way that maximized separation among classes [12]. Based on the cultivars, 23 apple varieties were grouped into cultivar. Golden Delicious (c. GD), cultivar. Jonathan (c. JT), cultivar. Ralls (c. RA) and cultivar. Fuji (c.FJ) by their parentage and origins. The PLS-DA score plots over two years are shown in Figure 3e,f.

In the first year, there were seven components observed, and the R^2^X and R^2^Y were 0.720 and 0.871, respectively. Also, seven components were identified in the second year, with R^2^X = 0.871 and R^2^Y = 0.722. The total correct classification was 95.65% for both years. Only one variety from c.FJ was misclassified into c. GD and c. RA in the first and second year, respectively. There was a close relationship of c.FJ with these two cultivars, because their parents were Ralls and Red Delicious. These phenomena could also be detected in Figure 3e,f. The separations for c. RA (in blue) and c.FJ (in yellow) were not distinct. However, the separation was clearer than PCA, especially for c. GD and c. JT, proving that PLS-DA had a better capacity to discriminate apple varieties.

The VIP values for two years are indicated in Table 2. A total of 14 volatile compounds were higher than one, indicating the importance of identifying characteristic variables. Ethyl 2-methylbutyrate, 2-methyl-1-butanol, Z-3-Hexenyl acetate, E-2-hexen-1-ol, linalool and dodecanol were identified in both years. Furthermore, the values for 2-methyl-1-butanol were higher than 1.5 in both years, and were the most important variables.

### 3.3. Odor-Active Compounds

The odor thresholds in water and odor descriptions for volatile compounds are indicated in Table 2. The odor threshold information for propyl hexanoate, pentyl hexanoate, hexyl 2-butenoate, butyl octanoate and hexyl octanoate were unknown, so they were not taken into consideration. Moreover, if the OAV was greater than 1 among all the apple varieties, they would be labeled in Table 2, which can be regarded as apple characterized odor-active compounds for each year.

In the first year, a total of 6 volatile compounds were identified, including ethyl 2-methylbutyrate, hexanal, 1-hexanol, hexyl 2-methylbutanoate, E-2-nonenal and linalool, which are described as green (grass), fruity and floral (flower) notes. However, there were a total of 19 characterized odor-active compounds in the second year. 14 of these compounds were unique in the second year, consisting of ethyl butyrate, butyl acetate, butyl propionate, pentyl acetate, D-limonene, 2-methyl-1-butanol, E-2-hexenal, hexyl acetate, 6-methyl-5-hepten-2-one, hexyl propionate, 1-hexanol, nonanal, E-2-hexen-1-ol, 1-heptanol and naphthalene, which were regarded as green, fruity and sweet notes. Although the presence of naphthalene was uncertain, it was a characterized odor-active compound in the second year. A total of five compounds were detected in both years, which were ethyl 2-methylbutyrate, hexanal, 1-hexanol, E-2-nonenal and linalool, demonstrating that they were the most critical characterized odor-active compounds in the 35 apple varieties. Ethyl 2-methylbutyrate is frequently used as a food additive to obtain better product flavor. The toxicity values for hexanal, 1-hexanol, E-2-nonenal and linalool in LD50 rat oral were 4890 mg/kg, 4870 mg/kg, 5000 mg/kg and 2790 mg/kg, respectively. Their concentrations in all the apple varieties were much less than their toxicity values, proving all the 5 critical characterized odor-active compounds were safe and harmless for human smell and consumption.

Since the odor thresholds were taken into consideration, the results were different to volatile compound concentrations. Hexanal and 1-hexanol not only had high concentrations among all the apple varieties, the odor thresholds were also at high levels, especially for 1-hexanol (500 μg/L). They were the most potent odorant in the aldehyde and alcohol group, respectively, which were found in the apple varieties Golden Delicious and Braeburn [4,32], and also other fruits, such as mulberry [14], peach [48] and cranberry [49]. Although the quantity for butyl acetate, 2-methyl-1-butanol, E-2-hexenal, E-2-hexen-1-ol, hexyl 2-methylbutanoate and 1-heptanol were greater, the thresholds were also high. Thus, the OAV may not be higher than 1 for all apple varieties. For example, the threshold for E-2-hexen-1-ol could achieve 2319 μg/L, it was an odor-active compound only in the second year. However, lower concentration compounds contributed more to the characterized odor-active compounds, especially for ethyl 2-methylbutyrate. Because the threshold for ethyl 2-methylbutyrate was also low (0.13 μg/L), it was also considered as an odor-active compound in Fuji, Golden Delicious, Starking, Golden Reinders and Braeburn apples [3,4,5]. Nevertheless, ethyl 2-methybutyrate was the main OAV in pineapple [50] and cranberry [49]. In the cases of E-2-nonenal and linalool, they were similar and thresholds were both below one. Based on the literature, E-2-nonenal and linalool were not regarded as odor-active compounds in apple, but they were carried out in other fruit, such as mulberry [14], peach [48], cranberry [49] and strawberry [51].

**Figure 4:**
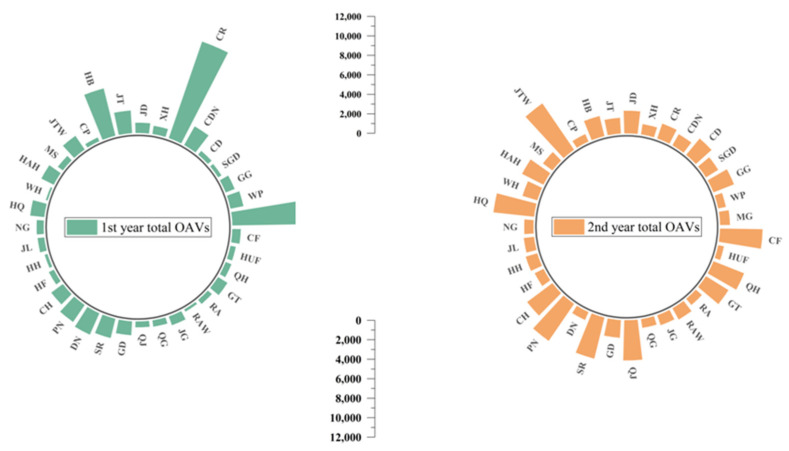
The sum of total OAVs for each apple varieties over two years. The apple cultivar abbreviation codes were described in Table 1.

Figure 4 indicates the total sum of odor active values (OAVs) for each apple variety over two years. The high total OAVs represent a rich aroma in apple. MG, CR and HB were revealed to have much higher total OAVs than other apple varieties in the first year. For MG, it was mainly because of the high concentration of E-2-hexenal, which was consistent with PCA results (Figure 3a,b). Regarding CR and HB, the concentrations for ethyl 2-methylbutyrate were high. There were six apple varieties that had higher total OAVs in the second year, including JTW, HQ, PN, SR, QJ and CF. Hexanal was the main reason leading to a high total OAV for JTW and QJ, which were probably aldehyde-like apples. The high OAV for HQ was mainly due to butyl acetate and E-2-nonenal. As for PN, SR and CF, butyl acetate, ethyl 2-methylbutyrate and linalool were dominant, respectively, in agreement with the PCA results.

For most of the apple varieties, the total OAVs in the first year were lower than the second year, which may mainly be caused by seasonal effects. Based on the Climate Communique in these two years from the government, where apples were harvested, a serious El Nino phenomenon was encountered in the first year, with serious drought and high temperatures in summer and autumn. The total amount of precipitation was 303.7 mm. In the second year, the phenomenon eased and the total amount of precipitation was 370.1 mm, which was 66.4 mm more than the first year, but there was still a drought in summer. Although the correlation between volatile compounds and climate data cannot be established statistically based on two harvest years’ data, seasonal effects such as temperatures and precipitation strongly impacted apple volatiles. More years or more production place data would be effective in further studying these relationships.

## 4. Conclusions

In the present work, 39 volatile compounds were identified and quantified among 35 apple varieties over two harvest years. The volatiles revealed different results over two years. Through PCA results, similarities and differences were found between apple varieties and years. However, it was not effective for discriminating apple varieties. PLS-DA results revealed a better capacity to differentiate apple cultivars based on their volatile profiles. The classification performances were accurate, especially in c. GD and c. JT. Moreover, it was also demonstrated that 2-methyl-1-butanol, *Z*-3-hexenyl acetate, dodecanol and ethyl 2-methylbutyrate were the most important variables for discriminating apple cultivars, especially 2-methyl-1-butanol. Taking odor thresholds into account, the most critically characterized odor-active compounds in apples were ethyl 2-methylbutyrate, hexanal, 1-hexanol, *E*-2-nonenal and linalool. In addition, the volatile concentrations and total OAVs among apple varieties between the two years were different, which implies that the seasonal factor greatly affects volatile formation in some varieties.

## Figures and Tables

**Table 1:** 35 apple varieties information.

No.	Name	Code	Reported Parentages or Origin	Harvest Date	No.	Name	Code	Reported Parentages or Origin	Harvest Date
1	Miguo	MG	unknown	9.28	19	Judeline	JL	Golden Delicious	9.28
2	White Pippin	WP	unknown	9.28	20	Huahong	HH	Golden Delicious	9.28
3	Grimes Golsen	GG	Golden Delicious	9.28	21	Hanfu	HF	Fuji	9.28
4	Stark Spur Golden Delicous	SGD	Golden Delicious	9.28	22	Changhong	CH	Fuji	9.28
5	Cloden	CD	Golden Delicious	9.28	23	Pinova	PN	Golden Delicious	9.28
6	Cardinal	CDN	unknown	9.28	24	Dounan	DN	unknown	9.28
7	Calville Rouge	CR	unknown	9.28	25	Starkrimson	SR	Golden Delicious	9.28
8	Xinhua	XH	Ralls	9.28	26	Golden Delicious	GD	Golden Delicious	9.28
9	Jonagold	JD	Golden Delicious/ Jonathan	9.28	27	Qiujin	QJ	Ralls/Golden Delicious	9.28
10	Jonathan	JT	Jonathan	9.28	28	Qinguan	QG	Golden Delicious	10.9
11	Hadi Bolaite	HB	unknown	9.28	29	Jiguan	JG	Golden Delicious	10.9
12	Clapp	CP	unknown	9.28	30	Ralls (weeping)	RAW	Ralls	10.9
13	Jonathan (New)	JTW	Jonathan	9.28	31	Ralls	RA	Ralls	10.9
14	Mutsu Spur	MS	Golden Delicious	9.28	32	Gongteng	GT	unknown	10.9
15	Hahong	HAH	unknown	9.28	33	Qihu 7	QH	unknown	10.9
16	Wenhong	WH	unknown	9.28	34	Huafu	HUF	Fuji	10.9
17	Hongqiaowang	HQ	unknown	9.28	35	Changfu 2	CF	Fuji	10.9
18	Ningguan	NG	Golden Delicious	9.28					

**Table 2:** List of identified volatile compound information, including retention time, retention index, CAS number, quantitative *m*/*z*, qualitative *m*/*z*, R^2^, odor description, threshold, OAV values and VIP values in PLS-DA over two years.

No.	Name	Retention Time (min)	Retention Index	CAS	Quantitative *m*/*z*	Qualitative *m*/*z*	*R* ^2^	Odor Descriptions	Thresholds (μg/L, in Water)	First Year		Second Year	
										OAV	VIP	OAV	VIP
1	Ethyl butyrate	5.889	1057	105-54-4	60	88/89	0.9993	oxidized apple, sweet	9 ^a^		0.70	>1	0.90
2	Ethyl 2-methylbutyrate	6.283	1073	7452-79-1	74	57/102	0.9993	fruity	0.13 ^b^	>1	1.17	>1	1.01
3	Butyl acetate	6.756	1094	123-86-4	61	43/56	0.9993	fruity, apple	66 ^b^		0.86	>1	0.72
4	Hexanal	6.922	1101	66-25-1	82	44/45	0.9916	grass	5 ^a^	>1	0.98	>1	1.42
5	Butyl propionate	8.603	1163	590-01-2	57	56/75	0.9982		25 ^c^		0.77	>1	1.11
6	Pentyl acetate	9.484	1196	628-63-7	43	70/61	0.9968	cooked apple, banana	43 ^a^		0.92	>1	0.72
7	D-Limonene	10.095	1218	5989-27-5	68	67/93	0.9901	citrus, mint	34 ^c^		0.72	>1	0.90
8	2-Methyl-1-butanol	10.492	1233	137-32-6	56	57/70	0.9922	wine	500 ^b^		1.73	>1	1.83
9	*E*-2-hexenal	10.628	1238	6728-26-3	98	80/83	0.9938	green, apple like	110 ^b^		0.93	>1	0.96
10	Butyl butyrate	10.76	1243	109-21-7	71	43/89	0.9962	fruity, berry	100 ^d^		0.95		0.76
11	Ethyl hexanoate	11.195	1259	123-66-0	88	70/99	0.9936	fruit	22 ^a^		0.61		1.07
12	Butyl 3-methylbutanoate	11.627	1274	109-19-3	85	56/103	0.9933	ethereal-fruity	17 ^d^		0.77		0.87
13	Hexyl acetate	12.278	1298	142-92-7	61	73/84	0.9962	sweet, fruity, floral	115 ^a^		0.97	>1	1.21
14	*Z*-3-hexenyl acetate	13.253	1334	3681-71-8	82	67/43	0.9990	green	13 ^a^		1.43		1.26
15	Pentyl butyrate	13.507	1344	540-18-1	71	70/89	0.9983	banana	210 ^a^		1.13		0.78
16	Propyl hexanoate	13.650	1349	626-77-7	99	61/117	0.9967	fruit	/	/	0.47	/	1.19
17	6-Methyl-5-hepten-2-one	14.014	1363	110-93-0	108	93/111	0.9951	mushroom, pepper	68 ^a^		0.89	>1	1.25
18	Hexyl propionate	14.091	1366	2445-76-3	75	57/84	0.9974	sweet	8 ^a^		1.12	>1	0.95
19	1-Hexanol	14.570	1384	111-27-3	56	43/55	0.9946	floral, green	500 ^b^	>1	1.25	>1	0.85
20	Nonanal	15.529	1421	124-19-6	98	81/82	0.9963	citus-like, floral	2.53 ^b^		1.12	>1	0.89
21	*E*-2-hexen-1-ol	15.967	1438	928-95-0	82	41/67	0.9996	green, walnut	2319 ^a^		1.12	>1	1.63
22	Butyl hexanoate	16.099	1443	626-82-4	99	87/117	0.9929	herbaceous	700 ^b^		0.83		0.70
23	Hexyl butyrate	16.167	1446	2639-63-6	89	69/84	0.9937	fruity	250 ^c^		0.97		0.79
24	Hexyl 2-methylbutanoate	16.467	1457	10032-15-2	103	74/87	0.9913	fruity, pungent	22 ^d^	>1	1.15		0.87
25	Ethyl octanoate	16.667	1465	106-32-1	88	57/101	0.9932	fruit, fat	19.3 ^a^		0.83		0.76
26	1-Heptanol	17.273	1489	111-70-6	70	55/56	0.9933	chemical, green	425 ^a^		1.13	>1	0.96
27	2-Ethylhexanol	18.146	1525	104-76-7	57	41/43	0.9939	rose, green	2548 ^a^		0.53		0.78
28	Pentyl hexanoate	18.636	1546	540-07-8	70	43/117	0.9979		/	/	0.94	/	1.18
29	*E*-2-nonenal	19.161	1568	18829-56-6	55	43/70	0.9911	fatty, green	0.69 ^e^	>1	1.13	>1	0.95
30	Linalool	19.529	1584	78-70-6	93	71/121	0.9991	flower, lavender	0.17 ^e^	>1	1.01	>1	1.18
31	Hexyl 2-butenoate	19.689	1590	19089-92-0	87	41/69	0.9970		/	/	0.93	/	0.65
32	1-Octanol	19.794	1595	111-87-5	56	55/70	0.9971	green	110 ^f^		0.98		1.01
33	Hexyl hexanoate	20.819	1650	6378-65-0	117	84/99	0.9973	green	6400 ^b^		0.72		0.65
34	Butyl octanoate	20.881	1653	589-75-3	145	127/101	0.9984	fruit	/	/	0.90	/	0.64
35	Estragole	21.837	1708	140-67-0	148	117/121	0.9997	licorice, anise	16 ^e^		0.78		0.75
36	Naphthalene	22.943	1783	91-20-3	128	64/102	0.9973	Tar, floral, fruity	6 ^a^		1.38	>1	0.79
37	Hexyl octanoate	23.963	1861	1117-55-1	84	56/145	0.9969	herb, green, oil	/	/	0.79	/	0.79
38	Anethole	24.180	1878	104-46-1	148	117/147	0.9984		50 ^a^		0.73		0.69
39	Dodecanol	25.883	2027	112-53-8	55	69/70	0.9968	fat, wax	16 ^a^		1.40		1.20

Threshold in water from reference: ^a^ [43]; ^b^ [31]; ^c^ [44]; ^d^ [45]; ^e^ [46]; ^f^ [47].

## Data Availability

The data presented in this study are available on request from the corresponding author.

## Supplementary Materials

The following supporting information can be downloaded at: https://www.mdpi.com/article/10.3390/foods11050690/s1, Table S1: Apple volatile concentrations for 1st year (μg/L); Table S2: Apple volatile concentrations for 2nd year (μg/L); Table S3: Correlation matrix of apple volatiles in 1st year; Table S4: Correlation matrix of apple volatiles in 2nd year.
