# Supplementary material for: Characteristic Volatiles and Cultivar Classification in 35 Apple Varieties: A Case Study of Two Harvest Years
Source: Foods. 2022 Feb 25;11(5):690. doi: 10.3390/foods11050690 (PMC8909895; doi:10.3390/foods11050690)
Supplement: Supplementary file 1 [file foods-11-00690-s001.zip › foods-1578825-supplementary.pdf]

Supplementary material 1

Table S1 Apple volatile concentrations for 1st year (μg/L)

	1 MG	2 WP	3 GG	4 SGD	5 CD	6 CDN	7 CR	8 XH	9 JD	10 JT	11 HB	12 CP	13 JTW	14 MS	15 HAH	16 WH	17 HQ	18 NG
Ethyl butyrate	439.57	296.71	50.29	30.00	67.22	1072.15	1959.42	106.31	1252.46	70.63	713.21	70.57	32.57	40.99	115.15	30.06	8.38	189.23
Ethyl 2-methylbutyrate	45.80	23.15	4.26	3.11	3.41	11.68	1224.45	8.64	52.71	69.02	643.22	5.59	22.30	0.98	29.83	4.39	0.63	32.19
Butyl acetate	599.52	172.65	3209.56	896.54	3557.20	51.64	963.75	212.85	195.52	3178.46	1205.51	493.69	88.70	568.26	3860.96	566.95	12656.58	530.37
Hexanal	28108.81	3780.90	3822.18	1331.08	1346.68	8770.11	5344.51	3135.88	671.18	1910.65	388.99	84.18	6644.74	2978.45	461.74	11.83	1698.46	1871.59
Butyl propionate	19.30	6.93	41.85	21.03	92.40	55.36	149.58	30.83	26.06	104.70	59.30	4.36	7.22	4.76	473.03	13.18	157.10	5.85
Pentyl acetate	66.57	2.37	155.35	48.63	106.23	1.17	35.59	1.60	1.31	87.66	29.17	7.31	9.79	16.11	173.48	47.30	382.12	3.69
D-Limonene	293.99	12.83	35.39	25.35	26.08	33.60	427.19	86.32	114.40	17.05	22.72	65.45	71.47	23.06	53.70	17.46	40.41	25.91
2-Methyl-1-butanol	31086.05	1956.29	342.57	410.33	679.49	1087.73	21159.88	1456.13	3988.01	42360.03	9436.05	6955.66	43682.06	123.53	14555.33	2747.13	1222.39	340.69
E-2-hexenal	162032.74	2674.29	3721.58	892.55	1333.68	11295.27	6396.68	3572.53	740.03	2333.36	644.53	434.90	2644.76	3805.54	882.62	241.18	8789.33	1092.81
Butyl butyrate	113.38	94.54	299.79	124.21	874.91	710.71	134.92	195.33	383.57	167.76	66.05	20.02	7.77	58.93	565.29	78.64	414.98	6.23
Ethyl hexanoate	17.82	36.36	4.88	7.18	3.07	23.62	67.62	3.99	24.18	8.68	87.14	11.83	3.64	0.80	12.70	4.35	3.23	7.31
Butyl 3-methylbutanoate	80.05	8.94	13.91	11.18	15.16	15.21	31.51	20.80	23.89	11.74	16.33	36.16	6.04	6.98	15.47	14.36	7.99	10.76
Hexyl acetate	443.42	91.18	1172.48	404.78	832.72	98.88	612.44	11.56	32.82	1474.08	226.75	79.25	41.89	196.81	1790.04	114.39	1997.75	26.59
Z-3-Hexenyl acetate	5.67	5.65	27.95	14.03	3.48	7.45	14.47	4.97	5.48	7.02	8.57	10.48	33.61	2.15	3.95	203.36	17.92	27.30
Pentyl butyrate	13.25	9.58	24.18	13.48	27.33	59.55	12.59	8.05	20.99	11.33	3.54	1.80	5.67	3.92	40.98	5.31	19.27	0.40
Propyl hexanoate	4.13	1.53	1.29	1.18	4.68	8.31	19.06	3.90	7.27	12.66	6.52	0.68	4.76	1.32	78.14	0.42	8.49	4.23
6-Methyl-5-hepten-2-one	144.06	31.95	131.46	46.99	199.36	724.43	183.85	415.12	2204.98	221.39	98.29	98.26	40.47	9.31	1356.78	73.32	40.52	41.51
Hexyl propionate	110.91	24.81	34.03	21.58	25.96	42.54	28.34	25.91	24.14	72.12	24.67	26.66	18.38	17.11	66.02	4.52	52.77	19.53
1-Hexanol	13161.56	6451.37	4748.28	4942.15	5517.05	6130.95	5266.93	6843.53	11108.04	10082.02	5729.38	8056.07	6213.61	1163.20	4864.08	6978.92	3543.62	2333.09
Nonanal	56.08	10.83	7.92	4.59	8.10	8.35	11.33	27.05	2.79	4.49	2.13	1.74	24.73	8.59	5.48	2.24	12.72	8.27
E-2-Hexen-1-ol	7342.06	3225.32	3388.76	2106.57	2288.80	5321.98	5288.39	5894.94	5304.81	2948.33	7283.78	9136.26	1139.69	1028.17	5875.62	11022.30	4806.38	1623.97
Butyl hexanoate	36.29	65.55	119.65	51.41	178.35	364.12	47.17	137.91	117.69	115.44	45.44	11.03	5.13	19.98	519.88	17.78	345.32	1.41

Hexyl butyrate	132.84	273.85	463.59	240.18	515.15	987.21	250.19	215.73	525.98	279.85	56.07	14.98	16.87	23.11	425.04	21.18	331.64	23.18
Hexyl 2-methylbutanoate	5308.30	8684.89	2017.26	1050.74	1665.52	2836.15	6990.38	974.06	2052.44	28307.42	2882.39	1913.61	3016.83	93.83	20713.78	1303.80	18611.45	72.75
Ethyl octanoate	20.77	5.56	5.80	5.66	4.65	6.59	8.13	3.30	3.91	4.88	7.06	1.85	1.06	0.70	2.21	0.89	1.44	0.61
1-Heptanol	276.20	34.29	24.73	24.68	28.60	58.13	79.75	50.31	89.65	164.12	62.65	169.02	59.11	7.26	339.41	51.37	57.82	10.15
2-Ethylhexanol	36.55	4.41	24.13	6.66	30.45	8.79	17.71	15.91	16.62	16.28	8.78	9.83	7.36	3.11	6.05	12.41	8.39	6.05
Pentyl hexanoate	19.84	12.79	11.03	6.60	10.97	51.95	7.06	15.18	14.48	10.75	7.70	6.57	1.23	2.66	50.91	6.49	36.22	2.03
E-2-Nonenal	28.90	6.20	4.95	3.13	3.77	12.36	9.99	21.96	19.29	7.80	9.03	56.88	11.54	3.88	5.44	16.45	7.40	3.58
Linalool	29.66	3.57	5.12	4.33	5.25	14.83	7.42	9.01	39.85	11.52	7.14	26.27	2.96	1.36	10.34	3.22	2.87	16.59
Hexyl 2-butenoate	5.35	1.75	1.94	1.70	2.99	2.76	2.14	2.11	3.84	1.23	1.39	0.48	0.25	0.13	2.09	0.54	0.80	0.10
1-Octanol	28.87	19.27	14.66	15.31	24.71	28.39	22.70	39.62	52.82	72.37	15.41	51.69	22.03	4.39	12.57	38.15	13.32	8.59
Hexyl hexanoate	76.88	151.75	90.08	41.22	116.74	362.76	54.11	146.08	131.48	161.48	20.98	8.79	8.02	6.08	490.91	9.16	374.60	1.99
Butyl octanoate	2.87	5.74	26.17	7.35	31.81	13.23	5.38	34.44	3.62	10.41	3.94	0.93	0.75	1.36	162.53	2.99	148.72	0.09
Estragole	21.17	3.31	40.13	30.10	10.56	6.64	20.12	8.23	6.77	5.63	4.44	2.44	2.79	0.83	36.10	0.68	122.33	0.63
Naphthalene	22.84	3.06	3.91	2.26	5.65	9.40	4.37	9.48	9.14	4.85	2.81	7.37	5.46	3.01	8.45	6.81	10.32	4.33
Hexyl octanoate	11.05	7.03	15.78	7.34	25.22	12.42	6.47	23.82	9.58	15.72	1.84	0.90	2.05	0.37	104.54	1.16	79.95	0.33
Anethole	2.14	0.43	0.92	0.76	0.54	1.40	0.84	0.62	0.93	1.70	0.54	0.79	0.57	0.17	0.86	0.24	2.57	0.22
Dodecanol	23.13	1.48	1.57	1.31	1.16	7.09	1.68	0.65	2.51	2.24	3.33	3.08	17.13	2.90	0.51	2.03	2.19	1.62

Table S1 (continued) Apple volatile concentrations for 1st year (μg/L)

	19 JL	20 HH	21 HF	22 CH	23 PN	24 DN	25 SR	26 GD	27 QJ	28 QG	29 JG	30 RAW	31 RA	32 GT	33 QH	34 HUF	35 CF
Ethyl butyrate	105.39	76.94	123.48	318.98	78.33	367.29	98.88	59.98	53.70	205.34	1306.77	108.85	231.46	186.68	59.98	69.61	94.50
Ethyl 2-methylbutyrate	8.13	3.30	13.80	187.06	13.45	34.35	24.08	5.11	5.30	32.83	39.24	7.19	26.87	43.14	9.97	17.18	17.58
Butyl acetate	2657.92	3101.34	693.28	2086.00	2923.48	2011.90	695.68	403.44	1928.24	1163.35	6533.85	395.49	959.04	2525.97	835.80	1463.11	4336.68
Hexanal	987.91	168.36	81.29	43.63	7203.50	10481.22	7804.73	4975.82	458.24	155.38	111.64	387.72	176.87	54.15	178.18	53.92	37.07

Butyl propionate	50.42	25.01	41.02	15.33	284.38	47.02	284.41	30.84	51.00	78.63	76.00	6.50	22.64	312.38	87.03	47.72	314.08
Pentyl acetate	112.31	115.14	11.26	33.27	119.65	80.67	45.19	10.10	88.04	22.71	157.99	2.38	5.21	92.46	45.78	43.96	240.44
D-Limonene	69.69	27.62	174.25	154.28	844.67	780.52	106.52	90.41	8.33	264.14	85.77	23.53	73.03	24.98	291.59	370.33	9.52
2-Methyl-1-butanol	9531.80	1173.64	17539.49	1806.29	4198.12	731.63	9144.71	6128.40	4681.25	7412.47	2980.77	7695.72	9853.43	18000.59	18452.46	8504.83	10150.00
E-2-hexenal	1278.88	253.76	195.28	61.52	4792.79	6592.72	5779.94	1381.57	1521.07	197.15	209.62	389.34	299.21	491.92	423.17	403.68	562.05
Butyl butyrate	252.71	22.28	85.04	15.22	304.07	78.72	93.63	478.52	106.27	370.75	557.60	9.35	34.90	160.28	108.58	145.04	352.02
Ethyl hexanoate	4.27	1.39	3.38	16.91	11.14	83.68	9.69	4.33	9.17	64.99	24.14	2.79	23.97	66.51	6.17	7.63	17.63
Butyl 3-methylbutanoate	12.93	12.09	15.10	12.62	41.81	44.02	12.60	12.26	7.97	10.55	13.25	10.87	13.26	12.21	7.95	9.50	9.72
Hexyl acetate	789.14	1199.88	46.48	156.12	1527.74	898.44	625.49	256.84	822.56	376.59	2752.13	34.88	44.52	653.97	273.96	390.77	1286.08
Z-3-Hexenyl acetate	8.98	22.30	6.44	123.83	13.87	16.10	2.33	2.25	4.86	31.52	5.36	3.91	5.42	3.43	25.33	60.40	9.11
Pentyl butyrate	12.97	1.66	10.73	1.08	15.00	4.61	19.93	34.77	6.91	19.23	20.11	0.88	3.83	18.32	10.59	13.46	20.82
Propyl hexanoate	11.93	1.79	1.64	0.90	11.67	7.68	65.36	5.61	4.32	6.96	5.39	1.41	1.96	30.52	6.80	4.94	11.45
6-Methyl-5-hepten-2-one	442.96	27.23	76.73	16.78	609.05	182.68	529.28	991.96	27.11	818.56	1383.65	83.70	340.98	293.68	1522.35	632.66	507.89
Hexyl propionate	32.56	30.75	38.47	31.83	68.59	21.14	36.82	47.01	35.38	32.76	53.95	34.30	53.48	73.78	30.37	38.73	74.29
1-Hexanol	4430.18	4251.43	5503.99	2397.58	4418.01	3275.37	4273.39	11446.78	5861.17	6916.74	9651.17	9161.50	10540.66	7985.53	7199.46	6947.44	10225.22
Nonanal	6.47	6.64	5.40	4.90	7.38	10.46	5.39	6.26	7.03	3.04	5.29	4.22	4.38	4.06	6.66	3.78	3.27
E-2-Hexen-1-ol	2712.50	4669.63	4122.85	969.46	426.65	544.08	3067.40	4005.98	16708.09	3833.94	5382.04	6991.85	8727.15	14627.15	6497.35	11565.57	16217.50
Butyl hexanoate	180.17	46.48	23.63	6.81	222.27	171.53	113.76	186.96	49.33	79.95	181.79	3.37	18.15	102.92	74.73	134.60	163.75
Hexyl butyrate	178.21	63.77	136.92	10.24	402.83	55.87	91.13	814.65	140.17	583.99	876.36	33.84	173.87	182.14	121.22	176.57	304.50
Hexyl 2-methylbutanoate	6927.94	3570.12	8436.19	261.12	10552.39	271.92	5839.00	6985.45	8711.39	5449.99	7075.02	1077.88	4551.89	14409.45	7442.33	6097.74	11369.38
Ethyl octanoate	1.10	0.54	0.51	1.28	1.79	13.04	2.28	1.26	0.77	2.20	4.72	0.50	1.79	3.41	1.00	1.66	1.89
1-Heptanol	52.19	35.74	57.64	16.94	69.58	44.62	103.48	69.15	45.67	52.56	80.22	53.83	94.37	122.08	120.32	125.21	90.65
2-Ethylhexanol	5.04	6.83	5.81	7.40	9.24	9.26	5.77	6.02	5.10	20.82	6.72	5.63	7.65	11.22	8.77	19.39	6.32
Pentyl hexanoate	15.87	6.66	6.94	2.22	11.97	11.35	11.85	23.02	9.11	8.49	15.40	4.39	7.39	16.96	10.05	24.50	15.77

E-2-Nonenal	4.59	2.33	2.57	1.79	4.48	4.91	3.02	4.98	3.82	2.19	3.53	5.02	4.34	4.87	6.71	10.33	8.85
Linalool	6.51	2.65	3.09	2.91	4.30	1.96	3.66	9.33	1.33	9.26	7.19	2.52	3.81	8.32	15.34	8.07	13.59
Hexyl 2-butenoate	0.49	0.21	0.38	0.10	2.04	0.24	0.53	8.77	0.37	2.57	5.36	0.20	0.68	1.24	1.52	0.75	1.83
1-Octanol	11.90	24.97	23.62	12.03	76.20	19.79	6.42	29.17	17.31	29.06	103.55	21.96	60.78	36.01	26.49	27.98	35.74
Hexyl hexanoate	172.06	58.72	46.59	4.96	202.84	139.56	116.37	307.71	75.36	67.40	369.03	26.91	98.19	129.09	68.15	188.84	126.45
Butyl octanoate	36.50	4.56	4.44	0.48	150.48	85.19	40.18	29.95	14.54	8.81	43.06	0.72	3.52	58.99	35.38	67.36	71.11
Estragole	3.64	2.18	4.31	0.92	298.88	82.42	74.09	289.51	43.50	2.64	2.29	2.18	32.10	2.69	34.18	2.04	5.54
Naphthalene	7.10	3.79	5.55	4.03	6.10	4.36	4.94	3.73	4.56	4.82	6.62	6.32	6.20	7.91	7.21	8.72	9.22
Hexyl octanoate	50.76	2.22	3.90	0.32	61.35	24.73	22.96	30.32	6.45	3.09	52.31	5.67	15.81	32.38	17.22	34.66	36.57
Anethole	0.47	0.31	0.35	0.23	3.52	1.00	0.90	3.62	1.20	0.41	0.35	0.31	0.79	0.44	0.70	0.38	0.39
Dodecanol	1.44	1.79	1.10	0.75	2.21	1.21	1.97	1.21	1.05	1.59	1.01	1.13	1.28	1.71	0.79	2.51	1.86

Supplementary material 2

Table S2 Apple volatile concentrations for 2nd year (μg/L)

	1 MG	2 WP	3 GG	4 SGD	5 CD	6 CDN	7 CR	8 XH	9 JD	10 JT	11 HB	12 CP	13 JTW	14 MS	15 HAH	16 WH	17 HQ	18 NG
Ethyl butyrate	317.89	1095.52	813.37	139.33	825.15	2107.34	2703.06	890.31	594.30	242.39	1448.42	73.61	323.89	368.22	2012.78	232.97	532.81	93.85
Ethyl 2-methylbutyrate	10.78	21.56	47.30	9.35	18.92	17.71	26.07	22.11	5.61	4.40	66.66	12.98	8.69	18.37	21.01	12.76	46.31	8.14
Butyl acetate	4577.10	481.14	10594.32	2265.12	5086.62	150.48	2036.10	733.92	8860.50	21073.80	6140.64	932.58	407.88	6266.70	15915.24	4962.54	60858.60	254.76
Hexanal	1505.88	722.54	2637.19	1754.69	5412.70	1058.66	1263.46	243.65	1516.66	196.82	239.11	140.76	22780.75	119.11	264.07	674.33	1980.31	780.26
Butyl propionate	297.11	319.81	235.74	176.99	344.98	301.18	271.54	331.78	411.40	343.95	362.74	285.43	244.29	281.80	251.71	451.22	708.84	416.10
Pentyl acetate	97.67	246.99	615.96	788.62	1382.11	239.41	306.07	279.97	2081.46	843.19	653.28	219.09	207.22	776.27	1824.18	775.79	3523.58	313.08
D-Limonene	852.00	984.00	5976.00	3264.00	1752.00	2064.00	2256.00	4656.00	7812.00	2760.00	1992.00	1020.00	4224.00	1728.00	3216.00	1740.00	5028.00	3084.00
2-Methyl-1-butanol	78815.00	33085.00	33445.00	18250.00	9215.00	18830.00	127255.00	13730.00	60510.00	111020.00	115985.00	17315.00	54490.00	7625.00	114615.00	13375.00	57085.00	2070.00
E-2-hexenal	10186.00	4224.00	15687.10	4576.00	13471.70	1613.70	3606.90	1713.80	2983.20	2172.50	2745.60	1532.30	34724.80	1857.90	2857.80	5926.80	6575.80	2136.20
Butyl butyrate	519.63	120.58	1053.15	1432.24	3976.62	2374.35	2489.73	2783.81	4452.88	4327.09	1562.37	831.93	247.24	1001.10	2546.98	549.13	4518.81	92.82
Ethyl hexanoate	17.85	12.60	168.70	18.55	52.50	32.55	56.70	220.85	86.45	31.85	122.85	22.75	33.60	45.15	131.25	45.85	67.20	25.20
Butyl 3-methylbutanoate	292.42	20.40	69.70	117.31	62.90	57.80	37.40	102.01	2781.36	68.00	2689.56	112.21	39.10	130.91	2730.36	195.51	3578.71	202.31
Hexyl acetate	3512.94	378.26	12013.12	13401.70	15036.06	528.24	6293.58	155.20	18284.48	17840.50	10856.46	1229.10	853.50	6826.48	17626.28	2998.76	18758.80	558.66
Z-3-Hexenyl acetate	14.19	20.51	47.99	78.86	21.00	21.62	13.64	18.68	27.21	14.33	10.31	36.65	33.65	30.09	15.13	86.68	12.92	33.73
Pentyl butyrate	12.39	14.16	30.09	56.05	86.73	50.15	75.52	24.78	43.07	27.14	67.26	18.29	19.47	29.50	60.77	28.32	77.88	9.44
Propyl hexanoate	24.29	2.51	7.79	9.78	42.77	10.62	40.67	25.86	32.97	118.97	267.82	35.44	1.93	151.27	207.64	199.41	272.31	14.18
6-Methyl-5-hepten-2-one	357.00	293.50	1410.50	1699.00	1202.50	6240.00	139.00	847.50	1134.00	988.50	3681.50	718.50	1738.00	1401.00	3265.50	4591.00	2593.50	817.50
Hexyl propionate	96.08	10.56	35.28	99.04	119.36	40.96	27.04	23.52	347.20	118.96	382.80	31.04	46.72	41.36	493.28	34.00	1578.32	22.32
1-Hexanol	17355.00	16800.00	39055.00	25150.00	13690.00	22135.00	18175.00	28350.00	34820.00	35005.00	23280.00	13765.00	30975.00	11685.00	28375.00	15095.00	39145.00	14105.00
Nonanal	28.46	41.52	80.43	99.78	54.83	35.09	14.62	55.99	28.26	17.56	16.98	27.80	97.66	34.00	31.32	67.60	76.41	48.88
E-2-Hexen-1-ol	10793.80	7444.00	14739.12	10942.68	9193.34	6997.36	5694.66	9826.08	9714.42	8002.30	11426.54	10421.60	5136.36	10607.70	9714.42	5992.42	9565.54	9863.30
Butyl hexanoate	462.00	28.00	658.00	574.00	1113.00	532.00	413.00	1379.00	1449.00	1162.00	1862.00	497.00	105.00	952.00	1918.00	546.00	3108.00	28.00

Hexyl butyrate	475.00	112.50	967.50	1187.50	1875.00	1675.00	2600.00	1362.50	3550.00	2935.00	1085.00	410.00	255.00	525.00	2032.50	312.50	6032.50	110.00
Hexyl 2-methylbutanoate	89.40	120.90	121.80	87.48	119.22	126.24	109.44	114.18	118.14	162.36	61.02	133.86	92.52	112.86	130.26	171.84	50.04	10.56
Ethyl octanoate	5.16	2.05	87.73	5.47	43.14	4.95	86.40	58.07	17.14	9.77	24.84	2.64	5.49	4.85	33.99	4.29	26.55	1.65
1-Heptanol	12175.00	14175.00	41975.00	35000.00	14975.00	21525.00	17400.00	49550.00	42050.00	53650.00	41825.00	35575.00	35175.00	28950.00	56700.00	109225.00	58950.00	27425.00
2-Ethylhexanol	89.40	120.90	121.80	87.48	119.22	126.24	109.44	114.18	118.14	162.36	61.02	133.86	92.52	112.86	130.26	171.84	50.04	10.56
Pentyl hexanoate	49.90	20.12	20.97	33.65	60.61	68.55	43.91	68.64	48.90	36.36	130.30	34.17	16.32	91.10	77.14	62.58	130.07	4.54
E-2-Nonenal	13.09	15.20	29.82	38.36	20.36	16.00	142.84	46.93	272.52	123.54	37.96	158.93	80.69	188.38	202.49	59.07	316.03	182.35
Linalool	10.11	12.47	39.52	61.51	29.86	52.59	8.23	31.82	43.73	31.60	60.88	28.86	28.67	50.23	68.61	93.81	85.68	28.39
Hexyl 2-butenoate	9.78	8.02	18.06	13.95	73.70	15.75	17.19	11.87	34.61	24.41	18.49	3.19	2.70	6.01	23.55	11.23	108.26	2.95
1-Octanol	122.31	127.82	305.92	218.93	90.08	165.47	169.14	313.31	319.35	417.37	151.69	193.26	228.69	127.51	109.51	156.72	178.76	104.23
Hexyl hexanoate	376.33	64.14	195.29	549.12	301.44	747.51	392.31	731.91	1442.48	1325.25	936.28	287.13	86.74	467.46	2320.51	533.17	4440.37	28.74
Butyl octanoate	100.18	6.27	39.45	79.01	49.51	24.11	11.09	204.76	319.41	214.84	220.32	51.32	5.56	123.38	306.67	218.96	1137.07	1.54
Estragole	26.88	11.36	695.36	276.16	87.36	56.48	13.28	33.92	499.52	24.00	55.84	5.28	10.40	2.72	144.96	3.52	1468.64	14.40
Naphthalene	22.30	15.87	42.08	29.24	25.41	23.98	28.29	41.32	52.38	37.12	32.90	32.91	45.73	27.43	54.07	25.22	56.61	24.75
Hexyl octanoate	3.99	4.13	12.64	7.88	11.39	4.34	4.00	3.43	10.10	7.23	3.61	5.47	7.90	4.24	4.98	7.05	15.16	3.44
Anethole	49.86	5.51	10.52	57.61	20.53	18.88	10.00	107.72	352.62	391.04	56.47	37.27	4.86	40.74	249.30	152.11	1537.17	3.10
Dodecanol	3.86	4.53	6.52	4.69	5.12	3.68	4.41	7.95	7.47	6.28	5.78	4.77	4.50	5.25	7.83	5.84	11.10	6.03

Table S2 (continued) Apple volatile concentrations for 2nd year (μg/L)

	19 JL	20 HH	21 HF	22 CH	23 PN	24 DN	25 SR	26 GD	27 QJ	28 QG	29 JG	30 RAW	31 RA	32 GT	33 QH	34 HUF	35 CF
Ethyl butyrate	127.16	80.87	166.73	146.89	484.20	256.23	2321.25	129.09	361.81	88.10	99.18	138.99	258.29	759.28	1750.20	162.42	1229.31
Ethyl 2-methylbutyrate	10.67	11.44	11.44	11.88	13.53	17.16	145.75	11.55	10.67	5.61	6.60	7.92	9.46	32.67	183.70	9.02	51.26
Butyl acetate	1796.52	23431.98	298.98	1764.18	79747.80	4001.58	32871.30	2764.08	2740.98	2198.46	3357.42	166.32	203.94	8659.86	11595.54	3124.44	9614.22
Hexanal	1135.99	52.08	2381.38	11590.75	3587.64	490.22	2214.05	2849.11	13840.90	1394.59	2205.00	2748.67	1414.56	568.32	481.66	171.29	783.34
Butyl propionate	415.84	406.81	392.35	583.33	536.17	393.56	827.71	578.29	339.74	419.81	334.84	451.36	411.26	662.76	657.61	308.67	1065.22

Pentyl acetate	281.50	1487.54	242.35	541.76	2953.08	522.32	2375.80	513.69	132.54	95.31	251.15	270.07	251.50	903.46	767.29	175.46	1835.09
D-Limonene	5664.00	4584.00	2928.00	3660.00	11172.00	3276.00	3324.00	5580.00	2208.00	1968.00	2376.00	1836.00	2856.00	5280.00	3612.00	2520.00	7356.00
2-Methyl-1-butanol	13530.00	12600.00	34395.00	8480.00	90450.00	3940.00	14720.00	6245.00	12400.00	2030.00	3530.00	62645.00	12270.00	100100.00	162740.00	9380.00	19155.00
E-2-hexenal	4838.90	1377.20	6857.40	29913.40	4994.00	2286.90	5254.70	9629.40	61113.80	8093.80	11523.60	7140.10	3319.80	1927.20	1897.50	966.90	2063.60
Butyl butyrate	227.29	1313.40	211.67	771.21	14008.39	510.09	4397.36	3135.15	477.99	327.05	355.68	367.82	217.74	1480.82	974.20	756.46	954.25
Ethyl hexanoate	26.60	40.25	19.95	38.50	109.20	37.80	316.05	32.55	86.10	23.45	17.50	31.50	58.45	96.60	87.50	39.90	185.85
Butyl 3-methylbutanoate	93.51	95.21	11.90	27.20	6008.15	45.90	6584.49	166.61	57.80	28.90	15.30	78.20	40.80	3959.53	2619.85	265.22	7521.24
Hexyl acetate	2808.66	16350.74	209.12	3633.18	25496.80	4767.30	16790.72	13262.32	2954.68	5797.18	10217.16	362.92	438.68	6089.46	4462.46	2554.80	6939.46
Z-3-Hexenyl acetate	86.48	98.99	8.66	92.80	12.10	74.25	19.25	36.73	10.47	84.69	17.92	15.47	12.36	61.82	49.51	76.59	20.39
Pentyl butyrate	13.57	25.96	11.21	36.58	159.30	24.19	256.65	108.56	28.32	9.44	12.98	19.47	10.03	33.63	28.91	26.55	18.29
Propyl hexanoate	5.85	13.59	6.70	23.42	65.44	7.48	784.80	9.61	28.44	26.53	1.72	19.09	2.04	91.68	61.78	9.03	87.02
6-Methyl-5-hepten-2-one	1855.00	1438.00	215.50	1892.50	1223.50	2445.00	1646.50	1358.50	2564.00	129.00	889.50	580.00	451.00	3181.50	1045.00	1000.50	4527.00
Hexyl propionate	17.44	154.80	34.48	19.52	679.52	17.76	2616.40	136.56	105.28	24.32	58.72	111.44	26.72	168.64	95.44	19.44	265.68
1-Hexanol	10935.00	14615.00	10565.00	5855.00	59390.00	5725.00	32670.00	21105.00	12885.00	7645.00	9765.00	14205.00	13495.00	27965.00	22575.00	5475.00	51315.00
Nonanal	51.76	59.51	26.26	78.61	85.67	44.40	65.96	80.76	67.12	24.47	35.42	54.90	46.96	53.23	36.89	30.94	73.04
E-2-Hexen-1-ol	8746.70	8895.58	4466.40	4354.74	8746.70	3312.58	12840.90	7816.20	12803.68	3424.24	5210.80	9788.86	8523.38	4354.74	5508.56	4354.74	2754.28
Butyl hexanoate	140.00	987.00	49.00	1246.00	7147.00	756.00	6195.00	1330.00	1071.00	168.00	224.00	371.00	84.00	1456.00	931.00	770.00	1134.00
Hexyl butyrate	127.50	1480.00	232.50	427.50	16502.50	312.50	4350.00	3660.00	465.00	245.00	800.00	1032.50	487.50	1055.00	582.50	340.00	897.50
Hexyl 2-methylbutanoate	8.94	19.14	7.74	10.08	316.20	7.74	122.88	22.62	14.94	10.32	11.28	12.42	7.44	68.64	38.64	6.78	80.15
Ethyl octanoate	2.48	14.06	3.18	5.47	58.26	5.20	77.10	3.75	32.77	2.70	3.44	13.04	3.05	21.04	18.27	3.52	69.91
1-Heptanol	15225.00	20475.00	16300.00	8300.00	74325.00	11175.00	41950.00	30800.00	16125.00	9200.00	10350.00	26175.00	26175.00	72800.00	49700.00	10500.00	183500.00
2-Ethylhexanol	8.94	19.14	7.74	10.08	316.20	7.74	122.88	22.62	14.94	10.32	11.28	12.42	7.44	68.64	38.64	6.78	240.48
Pentyl hexanoate	7.39	75.97	6.18	168.96	125.21	121.29	276.81	73.39	114.95	11.10	20.10	47.98	8.97	114.97	68.57	77.03	118.96
E-2-Nonenal	21.98	22.20	9.41	22.72	210.13	11.68	189.49	98.21	22.63	116.94	15.97	88.51	13.67	73.00	47.54	19.02	82.79
Linalool	43.97	30.54	15.65	28.50	43.16	41.71	38.70	48.49	49.42	15.85	23.78	22.87	21.21	164.59	85.82	27.55	313.76

Hexyl 2-butenoate	3.68	16.13	3.20	4.35	79.03	3.62	68.23	56.69	7.49	3.97	8.61	4.45	4.12	11.52	7.45	3.88	22.89
1-Octanol	76.33	103.05	82.56	52.54	893.49	56.81	105.89	146.04	48.41	29.60	53.54	128.43	134.85	153.86	102.37	49.40	276.88
Hexyl hexanoate	110.99	2249.56	52.16	533.18	4326.10	380.03	3306.48	1098.57	1067.23	94.37	457.35	1273.60	286.24	1691.20	635.43	300.46	1740.05
Butyl octanoate	9.74	205.46	6.23	95.13	1010.03	41.92	571.86	202.66	461.64	10.19	29.36	53.48	13.33	481.69	123.42	62.33	312.55
Estragole	14.88	166.40	21.28	37.12	593.76	14.40	1065.60	33.12	75.04	2.40	3.36	8.96	12.32	8.96	17.60	3.20	37.28
Naphthalene	27.19	46.71	22.29	35.58	86.72	36.27	47.88	42.70	29.84	14.30	18.89	21.19	23.66	54.25	60.78	19.46	94.79
Hexyl octanoate	4.12	4.97	2.68	4.49	12.99	4.98	13.44	5.59	5.37	2.31	3.18	4.51	3.69	6.18	6.03	2.41	11.32
Anethole	14.30	300.56	5.60	14.09	658.76	8.35	140.58	119.78	436.65	4.26	25.00	81.09	34.20	189.63	40.43	11.93	158.46
Dodecanol	5.71	6.28	8.46	8.80	52.00	7.64	7.53	7.34	7.98	8.27	11.60	7.18	20.05	7.34	20.30	7.32	17.10

Supplementary material 3

Table S3 Correlation matrix of apple volatiles in 1st year

	1	2	3	4	5	6	7	8	9	10	11	12	13	14	15	16	17	18	19	20	21	22	23	24	25	26	27	28	29	30	31	32	33	34	35	36	37	38	39
1	1.0000	0.6890	-0.0626	0.1456	-0.0329	-0.1484	0.1667	0.0202	0.0860	0.2128	0.4992	0.2483	0.0702	-0.1243	0.2254	0.0113	0.3443	-0.0101	0.1716	0.0320	-0.0305	0.0605	0.3837	-0.1111	0.3886	0.0208	0.1416	0.1265	0.0787	0.3257	0.3295	0.2691	0.1520	-0.1814	-0.1626	0.0806	-0.0912	-0.0512	0.0627
2		1.0000	-0.0902	0.0334	0.0666	-0.1197	0.1912	0.1924	-0.0068	-0.1334	0.5588	0.1620	-0.0542	-0.0085	-0.1094	0.0748	-0.1114	-0.1089	-0.0913	-0.0077	-0.0044	-0.1565	-0.0856	-0.0149	0.2607	-0.0106	0.1199	-0.1450	0.0045	-0.0214	0.0208	-0.0943	-0.1774	-0.1618	-0.0898	-0.1653	-0.1709	-0.0625	-0.0258
3			1.0000	-0.1737	0.3744	0.9360	-0.0503	-0.1298	-0.0756	0.3932	-0.0874	-0.1384	0.8183	-0.0709	0.1717	0.1211	0.0084	0.3262	-0.1421	-0.0834	0.0389	0.5179	0.2287	0.5171	-0.0825	0.0496	-0.0238	0.3518	-0.2117	-0.2085	0.0102	0.1460	0.5305	0.6368	0.1814	0.1235	0.6714	0.2437	-0.1857
4				1.0000	-0.0538	-0.0704	0.3789	0.3005	0.8728	-0.0157	0.0897	0.7919	-0.0515	-0.1626	0.1461	0.0394	-0.1161	0.4443	0.2082	0.8351	-0.2008	0.0339	0.0288	-0.0916	0.8043	0.3271	0.4661	0.1703	0.2438	0.2763	0.3447	-0.0693	0.0376	-0.0001	0.2159	0.6151	-0.0450	0.4381	0.7788
5					1.0000	0.5163	0.1411	0.1410	-0.0921	0.3417	0.1329	-0.0087	0.5294	-0.2060	0.4333	0.8262	0.2829	0.5090	-0.0489	-0.1631	0.2196	0.6171	0.1643	0.6187	-0.0811	0.5052	-0.0934	0.4672	-0.1968	-0.0445	0.0280	0.0501	0.4959	0.7385	0.2538	0.1272	0.7077	0.1905	-0.1788
6						1.0000	-0.0220	-0.0843	0.0167	0.3813	-0.1174	-0.0565	0.8229	-0.0691	0.2330	0.2232	0.0058	0.4124	-0.1148	-0.0172	0.1550	0.5595	0.1763	0.5358	-0.0152	0.1517	0.0165	0.3931	-0.1860	-0.1641	0.0040	0.0458	0.5013	0.7029	0.2031	0.2109	0.7071	0.2593	-0.1100
7							1.0000	0.0285	0.1625	-0.0729	0.3431	0.5657	0.1138	0.0084	-0.0843	0.0046	0.1268	0.1275	-0.1241	0.1492	-0.2578	0.0874	-0.0285	-0.0452	0.3339	0.0689	0.1807	-0.0417	-0.0295	-0.0169	0.0372	0.1864	0.0323	0.3961	0.4766	0.0934	0.1663	0.3662	0.0571
8								1.0000	0.3329	-0.2175	0.0313	0.1779	-0.0114	-0.1180	-0.0752	0.1794	-0.0236	0.4141	0.3904	0.3811	0.0515	-0.1431	-0.1946	0.4731	0.2112	0.5093	0.2136	-0.0663	0.1378	0.1697	0.0152	0.1502	-0.1026	-0.1130	-0.1367	0.2901	-0.0509	0.0968	0.5691
9									1.0000	-0.0552	-0.0010	0.7774	-0.0322	-0.0898	0.0288	-0.0464	-0.1019	0.5799	0.3780	0.8560	0.0421	-0.0717	-0.0602	-0.0176	0.7632	0.4770	0.5803	0.1367	0.3353	0.4170	0.3455	-0.0250	-0.0355	-0.0843	-0.0047	0.8025	-0.0511	0.2926	0.7913
10										1.0000	-0.0639	-0.0616	0.4446	-0.2280	0.8428	0.2109	0.4835	0.2286	0.1418	-0.0861	-0.0618	0.7494	0.8571	0.2217	0.0600	0.1190	0.2756	0.6466	-0.1255	0.1454	0.5771	0.2106	0.7109	0.3999	0.2179	0.1689	0.5325	0.2938	-0.1092
11											1.0000	0.2351	-0.0341	-0.1093	-0.0323	0.0907	0.0147	0.0037	0.0116	-0.0852	0.0845	-0.0373	0.0289	-0.0223	0.4494	0.0076	0.0916	-0.0340	-0.0540	0.0194	0.0368	0.0111	-0.0590	-0.0059	-0.0950	-0.1217	-0.1097	-0.1145	-0.0464
12												1.0000	0.0068	-0.1036	-0.0463	-0.0276	-0.0252	0.4340	0.2756	0.6429	-0.0674	-0.0188	-0.0665	-0.1084	0.7825	0.4410	0.5305	0.0480	0.5083	0.4623	0.2883	0.1917	-0.0455	0.0800	0.1984	0.6260	0.0046	0.3820	0.5442
13													1.0000	-0.1859	0.2719	0.3175	0.1888	0.4530	-0.0392	-0.0837	0.0017	0.5707	0.3669	0.5800	0.0536	0.2381	-0.0033	0.3424	-0.2574	-0.1765	0.1732	0.3776	0.6132	0.6484	0.2383	0.0691	0.7142	0.2656	-0.1677
14														1.0000	-0.2411	-0.1969	-0.1697	-0.3317	-0.1645	-0.1357	0.0820	-0.2350	-0.2650	-0.2325	-0.1841	-0.1742	0.0460	-0.1944	0.0329	-0.1707	-0.2410	-0.0584	-0.2728	-0.1363	-0.1395	-0.0588	-0.2112	-0.2274	-0.0354
15															1.0000	0.4111	0.4899	0.3173	0.1609	-0.0441	0.0039	0.7851	0.8334	0.2790	0.1190	0.2740	0.1520	0.8222	-0.1005	0.1931	0.5502	0.0388	0.7458	0.3613	0.2441	0.2190	0.4702	0.3594	0.0235
16																1.0000	0.3090	0.2923	-0.1188	-0.0988	0.0346	0.5447	0.0667	0.4626	-0.0339	0.5794	-0.1304	0.4643	-0.1486	-0.0079	-0.0067	-0.1609	0.4530	0.5121	0.1014	0.0710	0.5633	0.0527	-0.1046
17																	1.0000	0.1452	0.3872	-0.1814	0.0393	0.4395	0.5647	0.1523	-0.0742	0.3441	0.0271	0.3973	0.0040	0.5245	0.5183	0.3835	0.5000	0.2583	0.1558	0.1933	0.3787	0.1444	-0.1453
18																		1.0000	0.5118	0.3867	0.2833	0.3449	0.2436	0.6136	0.3792	0.6554	0.2857	0.4196	0.0479	0.2652	0.3789	0.3528	0.4235	0.3974	0.2400	0.6479	0.4657	0.4749	0.3626
19																			1.0000	0.2285	0.4504	-0.0699	0.3064	0.1963	0.2708	0.4483	0.3343	0.1047	0.3645	0.5387	0.5952	0.5614	0.1271	-0.1893	0.0197	0.5070	-0.0341	0.2394	0.3146
20																				1.0000	-0.1045	-0.0648	-0.1095	-0.0811	0.6500	0.3056	0.5034	0.0820	0.3016	0.2481	0.2646	-0.0850	-0.0369	-0.0606	-0.0067	0.7007	-0.0401	0.2476	0.8139
21																					1.0000	-0.0522	-0.0850	0.1847	-0.0894	0.2752	0.0215	0.1382	0.2459	0.1542	-0.0306	0.1318	-0.0076	0.0175	-0.2214	0.3103	0.0258	-0.1480	-0.0381
22																						1.0000	0.6065	0.4918	0.0338	0.3818	-0.0309	0.8989	-0.1297	0.0310	0.2936	0.0524	0.9207	0.7830	0.3341	0.2232	0.8486	0.3975	-0.1397
23																							1.0000	0.1817	0.0901	0.0376	0.1610	0.5844	-0.1378	0.1879	0.7351	0.3869	0.7041	0.2110	0.3054	0.0779	0.3574	0.3905	-0.0934
24																								1.0000	-0.0668	0.5259	-0.0549	0.4341	-0.1512	-0.0200	0.0683	0.2508	0.5371	0.5257	0.1853	0.1472	0.5901	0.3619	-0.0927
25																									1.0000	0.3283	0.5917	0.1486	0.2457	0.3273	0.3539	0.0287	0.0343	-0.0586	-0.0502	0.5066	-0.0760	0.2039	0.5593
26																										1.0000	0.2713	0.4888	0.3943	0.4689	0.2413	0.1897	0.3891	0.3469	-0.0103	0.6144	0.4468	0.2112	0.3489
27																											1.0000	0.0407	0.2870	0.3910	0.3419	0.1052	-0.0907	-0.0968	-0.1225	0.5237	-0.0931	0.1182	0.4170
28																												1.0000	0.0360	0.1863	0.3407	-0.0168	0.8756	0.5839	0.1879	0.4310	0.6764	0.3655	0.0639
29																													1.0000	0.6381	0.0836	0.2246	-0.1396	-0.1696	-0.1500	0.4745	-0.1698	0.0687	0.3678
30																														1.0000	0.3925	0.2538	0.0328	-0.1450	-0.1312	0.5745	-0.0770	0.1271	0.3411
31																															1.0000	0.3438	0.4612	0.0433	0.4292	0.3051	0.2042	0.5335	0.1980
32																																1.0000	0.2484	0.0840	0.1526	0.1585	0.1621	0.2298	-0.0355
33																																	1.0000	0.6903	0.3690	0.2392	0.8308	0.4505	-0.1229
34																																		1.0000	0.5148	0.1869	0.9143	0.4372	-0.1926
35																																			1.0000	-0.0650	0.4056	0.8752	-0.0884
36																																				1.0000	0.2556	0.2475	0.6400
37																																					1.0000	0.3832	-0.1768
38																																						1.0000	0.1952
39																																							1.0000

Supplementary material 4

Table S4 Correlation matrix of apple volatiles in 2nd year

	1	2	3	4	5	6	8	9	10	11	12	13	14	15	16	17	18	19	20
1	1.0000	0.5890	0.0989	-0.1630	0.1687	0.2303	0.4901	-0.1914	0.1848	0.5484	0.4199	0.1380	-0.3742	0.4640	0.4691	0.3133	0.3817	0.3160	-0.2290
2		1.0000	0.2259	-0.1375	0.5070	0.2931	0.4044	-0.1440	0.0712	0.5830	0.5274	0.1318	-0.0953	0.4433	0.5880	0.1016	0.5278	0.2607	0.0083
3			1.0000	-0.0750	0.3775	0.8528	0.2799	-0.1247	0.8301	0.3175	0.6245	0.7369	-0.1851	0.5916	0.4143	0.0604	0.6360	0.6683	0.2925
4				1.0000	-0.0934	-0.1215	-0.0965	0.8514	-0.0559	-0.0803	-0.1355	-0.1418	-0.0345	-0.0007	-0.1251	0.0081	-0.0467	0.0110	0.5055
5					1.0000	0.5209	0.0357	-0.1158	0.1993	0.4623	0.7650	0.1796	-0.0089	0.3576	0.4356	0.2700	0.5130	0.4005	0.2736
6						1.0000	0.2116	-0.1950	0.7072	0.4538	0.7543	0.7996	-0.1739	0.6118	0.5364	0.2245	0.7236	0.6914	0.3066
8							1.0000	-0.1721	0.2897	0.1133	0.3124	0.2652	-0.3282	0.1087	0.1249	-0.0060	0.0793	0.3952	-0.3052
9								1.0000	-0.1450	-0.0492	-0.2060	-0.1468	-0.0537	-0.0610	-0.1304	0.0231	-0.0804	-0.1186	0.3963
10									1.0000	0.3124	0.5284	0.7394	-0.3004	0.6731	0.2590	0.0144	0.4436	0.6811	0.2065
11										1.0000	0.6755	0.3026	-0.2573	0.5802	0.6676	0.2140	0.6309	0.5586	0.1998
12											1.0000	0.4922	-0.2488	0.5787	0.5927	0.3311	0.6708	0.7349	0.2046
13												1.0000	-0.1151	0.6115	0.3894	0.0422	0.5229	0.5920	0.1872
14													1.0000	-0.2279	-0.1938	0.0824	-0.2542	-0.3789	0.2277
15														1.0000	0.7446	0.0877	0.8139	0.4355	0.2730
16															1.0000	0.2312	0.8933	0.2898	0.0381
17																1.0000	0.1153	0.2524	0.1959
18																	1.0000	0.4346	0.2103
19																		1.0000	0.3798
20																			1.0000

	21	22	23	24	25	26	27	28	29	30	31	32	33	34	35	36	37	38	39
1	0.0803	0.2932	0.1199	0.3129	0.6109	0.1895	0.3396	0.3669	0.1596	0.1866	0.2090	0.0252	0.2218	0.1177	0.1918	0.2561	0.1843	-0.0438	-0.0135
2	0.1042	0.3790	0.0581	0.0332	0.3907	0.2343	0.0733	0.4921	0.0922	0.2607	0.2241	-0.0659	0.2604	0.2240	0.3427	0.3755	0.3298	0.0346	0.1581
3	0.1874	0.8461	0.8833	0.5180	0.3613	0.3087	0.4788	0.4564	0.5751	0.1176	0.7692	0.6526	0.8986	0.8831	0.7355	0.6257	0.6790	0.7827	0.6873
4	-0.0438	-0.0243	-0.0285	-0.0699	-0.0239	-0.1510	-0.0905	0.0536	-0.1154	-0.1289	-0.0338	-0.0107	-0.0902	0.0020	-0.0259	0.0333	0.1303	0.0059	-0.0148
5	-0.2780	0.4441	0.2468	-0.0689	0.2797	0.6180	0.1623	0.5870	0.2430	0.6908	0.4033	0.0786	0.5211	0.5029	0.3348	0.6385	0.4459	0.3236	0.3565
6	0.1844	0.7715	0.7085	0.4428	0.4187	0.4756	0.4866	0.5437	0.6816	0.3527	0.8324	0.4727	0.8971	0.8446	0.7872	0.7148	0.8062	0.7578	0.4665
8	0.0076	0.2028	0.2676	0.3105	0.2430	0.1863	0.2542	0.0446	0.2402	0.0964	0.1054	0.3094	0.2675	0.2422	0.0489	0.3506	0.0956	0.1969	0.2285
9	0.1402	-0.0677	-0.1120	-0.1787	0.0311	-0.2087	-0.1980	0.0963	-0.2110	-0.1291	-0.0747	-0.1477	-0.1092	0.0445	-0.0245	-0.0903	0.0530	0.0623	-0.0726
10	0.1902	0.8391	0.9689	0.7186	0.4188	0.2368	0.6416	0.3619	0.5184	0.0013	0.7443	0.8249	0.7285	0.7122	0.5048	0.5659	0.5908	0.5298	0.7063
11	0.3298	0.6085	0.2560	0.2973	0.7754	0.4589	0.3880	0.6267	0.2319	0.3584	0.3432	0.2486	0.4622	0.4195	0.4712	0.5399	0.4914	0.1255	0.1991
12	-0.0038	0.7403	0.5438	0.3638	0.5168	0.6916	0.5349	0.6413	0.4700	0.6589	0.5317	0.4233	0.7355	0.6997	0.5101	0.8188	0.6254	0.4077	0.5280
13	0.3026	0.6792	0.7014	0.4618	0.3739	0.2164	0.4201	0.3605	0.5293	0.0845	0.7502	0.5067	0.7229	0.6312	0.6146	0.5220	0.6578	0.5594	0.3733
14	-0.3204	-0.2336	-0.2858	-0.2944	-0.3245	-0.1024	-0.3109	-0.0245	-0.2890	0.0233	-0.3175	-0.2835	-0.2351	-0.2459	-0.1754	-0.1267	-0.1789	-0.2277	-0.1832
15	0.3152	0.8617	0.6531	0.4365	0.5157	0.1060	0.3692	0.7028	0.4129	-0.0190	0.7326	0.3206	0.6469	0.5659	0.6134	0.3639	0.5812	0.2782	0.3144
16	0.3129	0.6546	0.2372	0.2439	0.3761	0.2551	0.2300	0.7320	0.4290	0.1448	0.4786	-0.0096	0.5514	0.4869	0.5836	0.2342	0.4498	0.2729	-0.0074
17	-0.0940	0.1125	-0.0108	0.1612	0.0620	0.5152	0.2794	0.3752	-0.0413	0.6037	0.0959	0.0014	0.2341	0.2391	0.0622	0.3093	0.2026	0.1574	-0.0605
18	0.3241	0.7791	0.4645	0.2192	0.4138	0.1937	0.2072	0.7183	0.5468	0.1032	0.6999	0.1268	0.7510	0.6835	0.8208	0.3700	0.6310	0.5201	0.1746
19	0.2108	0.6233	0.6719	0.6545	0.5933	0.6845	0.7561	0.2730	0.4841	0.4846	0.5674	0.7981	0.6670	0.6534	0.5508	0.8247	0.7554	0.5045	0.5238
20	0.1361	0.2936	0.2775	0.1119	0.1899	0.2895	0.1654	0.2429	-0.0016	0.2537	0.3260	0.2709	0.3011	0.3417	0.3671	0.3970	0.5459	0.2518	0.2509
21	1.0000	0.2724	0.1634	0.2602	0.2880	-0.1436	0.1270	0.1052	0.2324	-0.2827	0.2635	0.1690	0.2102	0.2108	0.4306	-0.0551	0.3065	0.1914	-0.0988
22		1.0000	0.8439	0.5531	0.4957	0.2824	0.5103	0.7305	0.5097	0.1088	0.7296	0.5838	0.8438	0.8105	0.6654	0.6014	0.6357	0.5109	0.6216
23			1.0000	0.6431	0.3808	0.2334	0.5765	0.3485	0.5298	0.0048	0.7311	0.8122	0.7726	0.7587	0.5477	0.5736	0.5759	0.5856	0.7787
24				1.0000	0.4156	0.4015	0.9281	0.1495	0.3793	0.0685	0.4042	0.7891	0.3934	0.4001	0.2645	0.4036	0.5051	0.2170	0.4076
25					1.0000	0.3840	0.5033	0.3938	0.2206	0.2695	0.4135	0.3845	0.3901	0.3573	0.4402	0.5057	0.5611	0.1584	0.2505
26						1.0000	0.6508	0.2521	0.2670	0.8732	0.2312	0.4284	0.4050	0.4219	0.1595	0.7333	0.4899	0.2770	0.3319
27							1.0000	0.1950	0.3484	0.3753	0.3804	0.7745	0.4066	0.3990	0.2207	0.5772	0.5613	0.2020	0.4387
28								1.0000	0.2389	0.3004	0.4667	0.0607	0.6238	0.5962	0.4711	0.4497	0.4272	0.3140	0.1893
29									1.0000	0.0863	0.5583	0.3715	0.6196	0.6099	0.5910	0.4179	0.4832	0.6118	0.2154
30										1.0000	0.1129	0.1225	0.2838	0.3107	0.0454	0.6678	0.3534	0.1795	0.1730
31											1.0000	0.4070	0.7691	0.7676	0.7708	0.4674	0.7811	0.7141	0.3639
32												1.0000	0.4925	0.5080	0.2981	0.5988	0.5085	0.3631	0.7042
33													1.0000	0.9273	0.7362	0.6805	0.6532	0.8034	0.5207
34														1.0000	0.7394	0.6462	0.6684	0.8777	0.5392
35															1.0000	0.3962	0.7956	0.7269	0.2288
36																1.0000	0.6435	0.4546	0.5991
37																	1.0000	0.5818	0.3406
38																		1.0000	0.3354
39																			1.0000
